# Nimbolide, a neem limonoid inhibits cytoprotective autophagy to activate apoptosis via modulation of the PI3K/Akt/GSK-3β signalling pathway in oral cancer

**DOI:** 10.1038/s41419-018-1126-4

**Published:** 2018-10-23

**Authors:** Josephraj Sophia, Jaganathan Kowshik, Anju Dwivedi, Sujit K Bhutia, Bramanandam Manavathi, Rajakishore Mishra, Siddavaram Nagini

**Affiliations:** 1 Department of Biochemistry and Biotechnology, Faculty of Science, Annamalai University, Annamalainagar, 608 002 Tamil Nadu India; 2 Department of Biochemistry, School of Life Sciences, University of Hyderabad, Hyderabad, 500046 India; 3 Department of Life Science, National Institute of Technology Rourkela, Rourkela, 769008 Odisha India; 4 Centre for Life Sciences, School of Natural Sciences, Central University of Jharkhand, Ranchi, 835205 Jharkhand India

## Abstract

Of late, nimbolide, a limonoid from the neem tree (*Azadirachta indica*) has gained increasing research attention owing to its potent antiproliferative and apoptosis-inducing effects. The present study was designed to investigate the effect of nimbolide on autophagy and the time point at which the phosphorylation status of GSK-3β and PI3K dictate the choice between autophagy and apoptosis in SCC131 and SCC4 oral cancer cells. Additionally, we analysed changes in the expression of proteins involved in autophagy and apoptosis after therapeutic intervention with nimbolide in a hamster model of oral oncogenesis. Furthermore, we also demonstrate changes in the expression of key genes involved in apoptosis and autophagy during the stepwise evolution of hamster and human OSCCs. Nimbolide-induced stereotypical changes in oral cancer cells characteristic of both apoptosis and autophagy. Time-course experiments revealed that nimbolide induces autophagy as an early event and then switches over to apoptosis. Nimbolide negatively regulates PI3K/Akt signalling with consequent increase in p-GSK-3β^Tyr216^, the active form of GSK-3β that inhibits autophagy. Downregulation of HOTAIR, a competing endogenous RNA that sponges miR-126 may be a major contributor to the inactivation of PI3K/Akt/GSK3 signalling by nimbolide. Analysis of key markers of apoptosis and autophagy as well as p-Akt^Ser473^ during sequential progression of hamster and human OSCC revealed a gradual evolution to a pro-autophagic and antiapoptotic phenotype that could confer a survival advantage to tumors. In summary, the results of the present study provide insights into the molecular mechanisms by which nimbolide augments apoptosis by overcoming the shielding effects of cytoprotective autophagy through modulation of the phosphorylation status of Akt and GSK-3β as well as the ncRNAs miR-126 and HOTAIR. Development of phytochemicals such as nimbolide that target the complex interaction between proteins and ncRNAs that regulate the autophagy/apoptosis flux is of paramount importance in cancer prevention and therapeutics.

## Introduction

Oral squamous cell carcinoma (OSCC), the sixth most common malignancy worldwide with an annual incidence of over 300,000 newly diagnosed cases is one of the major public health problems due to the rising incidence of the disease especially among the younger population^1–3^. Despite significant progress in molecular diagnostics and therapeutics, the morbidity and mortality rates of OSCC are high and the 5-year survival remains low^4^. Novel molecularly targeted intervention strategies are therefore required to inhibit the development and progression of OSCC.

Recently, nimbolide, a major limonoid from the neem tree (*Azadirachta indica*) figured in the list of ten potential natural compounds for OSCC treatment based on large-scale mining as well as annotation of reliable compounds and bioactivity databases^5^. Extensive investigations by us as well as others provide ample evidence for the antiproliferative effects of nimbolide on a wide array of malignant cell lines in vitro^6–13^. Most importantly, nimbolide was shown to inhibit the development and progression of 7,12-dimethylbenz[a]anthracene (DMBA)-induced hamster buccal pouch (HBP) carcinomas that closely emulate human OSCCs in histology, precancerous lesions, propensity for invasion and metastasis and gene expression signatures^14–18^.

An overwhelming body of evidence indicates that nimbolide inhibits the proliferation of cancer cells by inducing apoptosis^11–13,19–26^. Nimbolide has been reported to transduce apoptosis by modulating signalling networks that regulate both the intrinsic and extrinsic pathways of apoptotic cell death^13,15,25,26^. Previously, we demonstrated that nimbolide exerts chemotherapeutic effects in the HBP model by inducing caspase-mediated apoptosis via targeting the phosphatidylinositol-3-kinase (PI3K) pathway with consequent activation of glycogen synthase kinase-3β (GSK-3β)^15^. More recently, Pooladanda et al., showed that nimbolide stimulates apoptosis of breast cancer cells through modulation of autophagy^27^. However, the underlying mechanism by which nimbolide toggles between apoptosis and autophagy remains to be elucidated.

Autophagy is an evolutionarily conserved form of programmed cell death (PCD) by which damaged and worn out organelles and proteins are packed into autophagosomes for degradation by lysosomes^28,29^. The intricate crosstalk between apoptosis and autophagy is mediated through the major players ATG5, Bcl-2, and Beclin-1^30–32^. Although recent studies have revealed an association between autophagy and clinicopathological features and prognosis of OSCC, the signalling pathways governing functional crosstalk between apoptosis and autophagy as well as the effect of nimbolide on these processes remain elusive.

In the present study, we investigated the effect of nimbolide on autophagy and the time point at which the phosphorylation status of GSK-3β and PI3K dictates the choice between autophagy and apoptosis in nimbolide treated SCC131 and SCC4 oral cancer cell lines. Additionally, we analysed changes in the expression of proteins involved in autophagy and apoptosis after therapeutic intervention with nimbolide in the HBP model. Furthermore, we also demonstrate changes in the expression of key genes involved in apoptosis and autophagy during the stepwise evolution of HBP carcinomas and human OSCCs.

## Results

### Nimbolide reduces the growth of SCC131 and SCC4 oral cancer cells

**Fig. 1:**
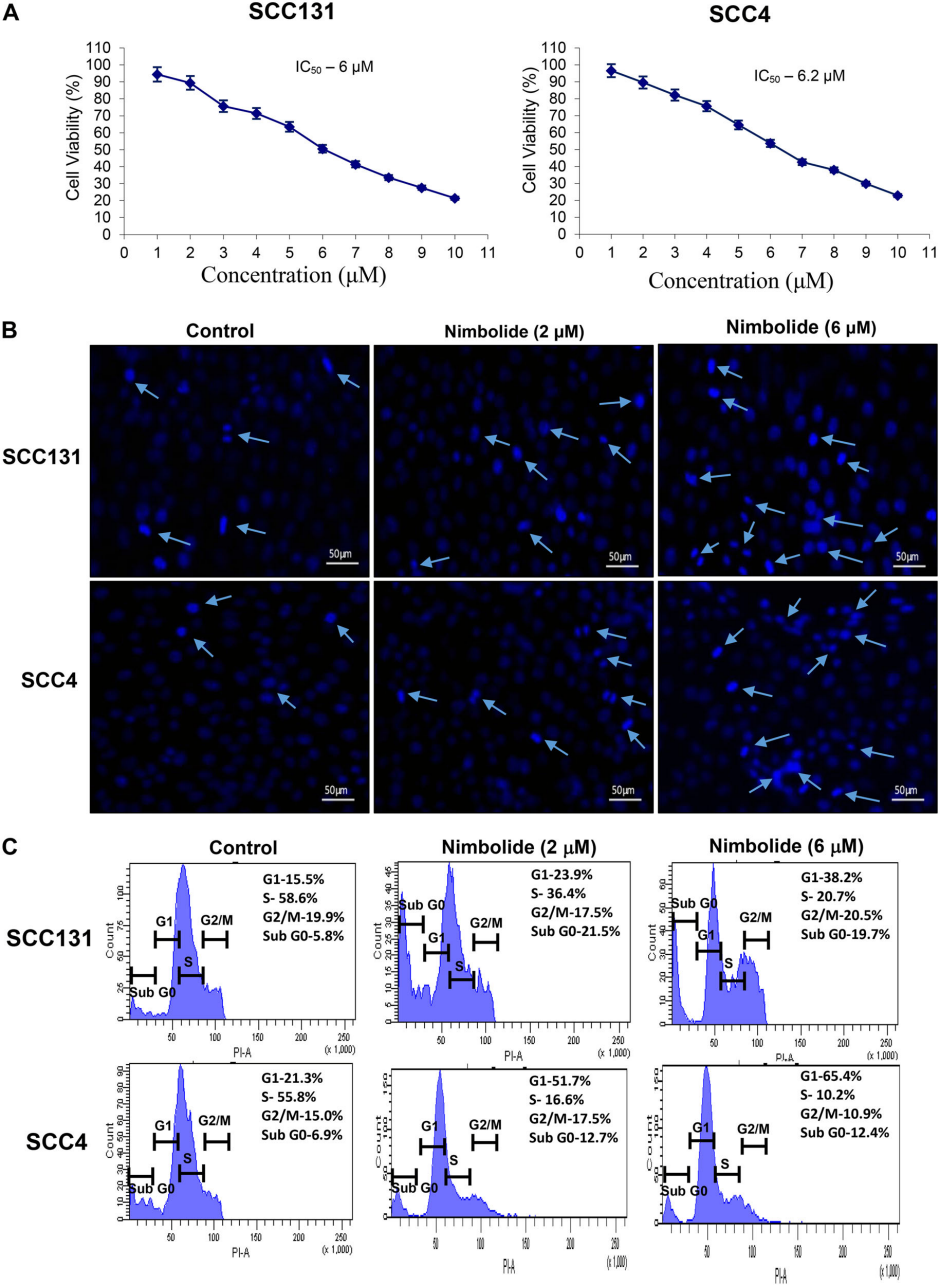
Nimbolide suppresses proliferation of SCC131 and SCC4 oral cancer cells. **a** IC_50_value of nimbolide in SCC131 and SCC4 cells is 6.0 and 6.2 μM respectively. **b** Morphological changes were evaluated by fluorescence microscopy using DAPI staining. DAPI staining shows apoptotic cells with chromosomal condensation and nuclear fragmentation and viable cells with intact DNA. **c** Cells were treated with nimbolide (2 and 6 µM) for 24 h. The DNA was stained with propidium iodide and cell-cycle distribution was analysed by flow cytometry. Sub G1 cells represent apoptotic cells with a lower DNA content. The data presented are representative of three independent experiments

We first determined the cytotoxic effects of nimbolide on oral cancer cells using MTT assay. When cells were treated with increasing concentrations of nimbolide (0–10 µM) for 24 h, the cell viability was significantly decreased in a dose-dependent manner. Nimbolide effectively attenuated the growth of SCC131 and SCC4 cells with IC_50_ values of 6 and 6.2 µM respectively (Fig. 1a).

### Nimbolide induces cytotoxicity via mitochondrial-mediated apoptosis

**Fig. 2:**
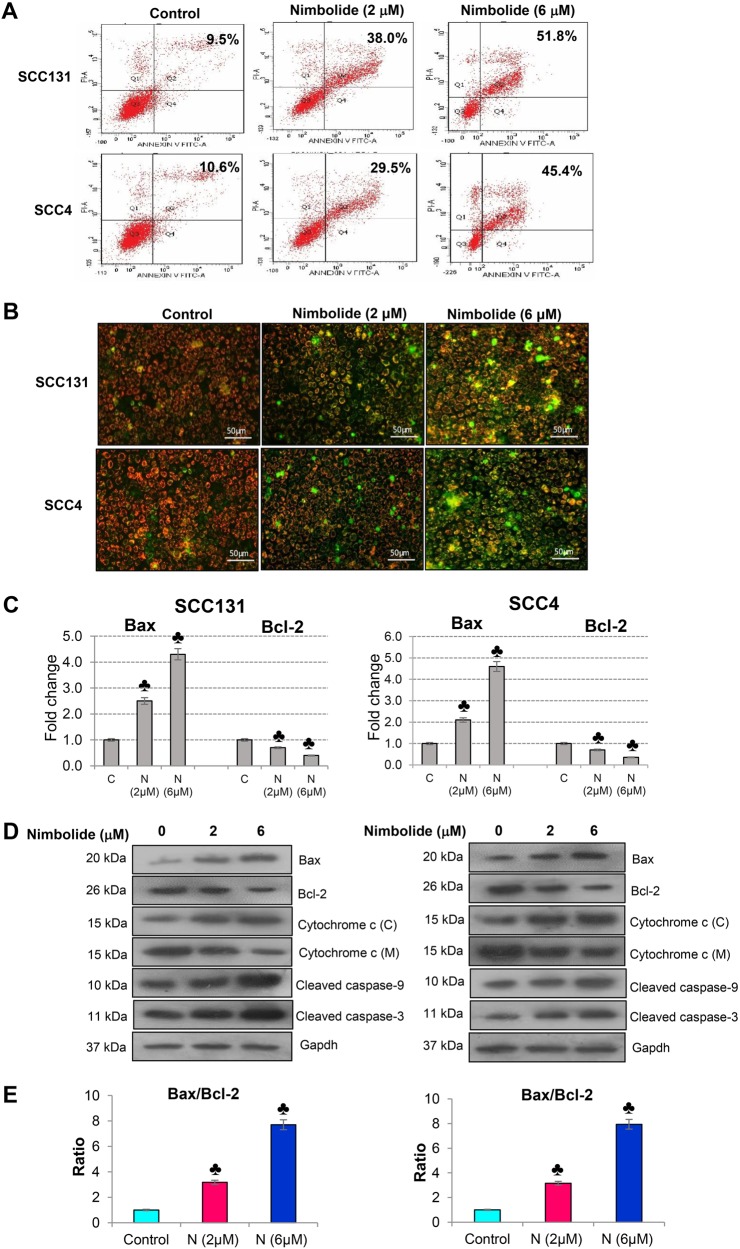
Nimbolide induces apoptosis in oral cancer cells. **a** A significant increase in the percentage of apoptosis after nimbolide treatment was determined using an Annexin V-FITC Apoptosis Detection Kit. The cells at early apoptosis are in the Q4 quadrant (Annexin V+/PI−), and the ones at late apoptosis are in the Q2 quadrant (Annexin V+/PI+). **b** Mitochondrial membrane potential was observed using JC-1 staining. Red fluorescence is visible in cell areas with high mitochondrial membrane potential, while yellowish green fluorescence of JC-1 monomer is seen in cell areas with low mitochondrial membrane potential. **c** Quantitative RT-PCR analysis of Bax and Bcl-2 in oral cancer cells treated with nimbolide for 24 h. The fold change in transcript expression for each gene was determined using the 2^−ΔΔCt^ method. Data are the mean ± SD of three independent experiments. Statistical significance was determined by the Mann–Whitney test (*p* < 0.05). **d** Immunoblot analysis of key molecules involved in apoptosis. Gapdh was used as loading control. **e** Bar graph representing Bax/Bcl-2 ratio in different groups. ♣ Significantly different from control (*p* < 0.05)

To assess whether nimbolide-induced cytotoxicity is caused by apoptosis, we examined the nuclear morphology of SCC131 and SCC4 cells using the fluorescent DNA-binding dye, DAPI. Cells treated with nimbolide for 24 h showed significant nuclear morphological changes characteristic of apoptosis (Fig. 1b). Analysis of the cell cycle by flow cytometry revealed significant increase in the subG1 cell population that represents apoptotic cells with a lower DNA content (Fig.1c). To quantify apoptosis, nimbolide treated cells were stained with annexin V. The percentage of apoptotic cells was decreased in untreated control cells, whereas both early and late apoptotic cells were increased in nimbolide treated cells (Fig. 2a).

As apoptosis is frequently associated with the collapse of the mitochondrial membrane potential (MMP), the ability of nimbolide to depolarize the mitochondrial membrane was investigated by JC-1 staining. In normal polarized mitochondria, JC-1 gets accumulated in an aggregated form seen as red punctate staining, whereas in cells with depolarized mitochondria, it gets distributed into the cytoplasm and appears as green diffuse monomeric staining. Treatment of cells with nimbolide induced a change in fluorescence from red to green indicating collapse of the MMP (Fig. 2b). Apoptosis induction in SCC131 and SCC4 cells upon nimbolide treatment was further confirmed by an increase in the Bax/Bcl-2 ratio and higher cytosolic cytochrome c relative to the mitochondrial fraction associated with increased expression of cleaved caspase -9 and -3 (Fig. 2c–e).

### Nimbolide induces autophagy in oral cancer cells

**Fig. 3:**
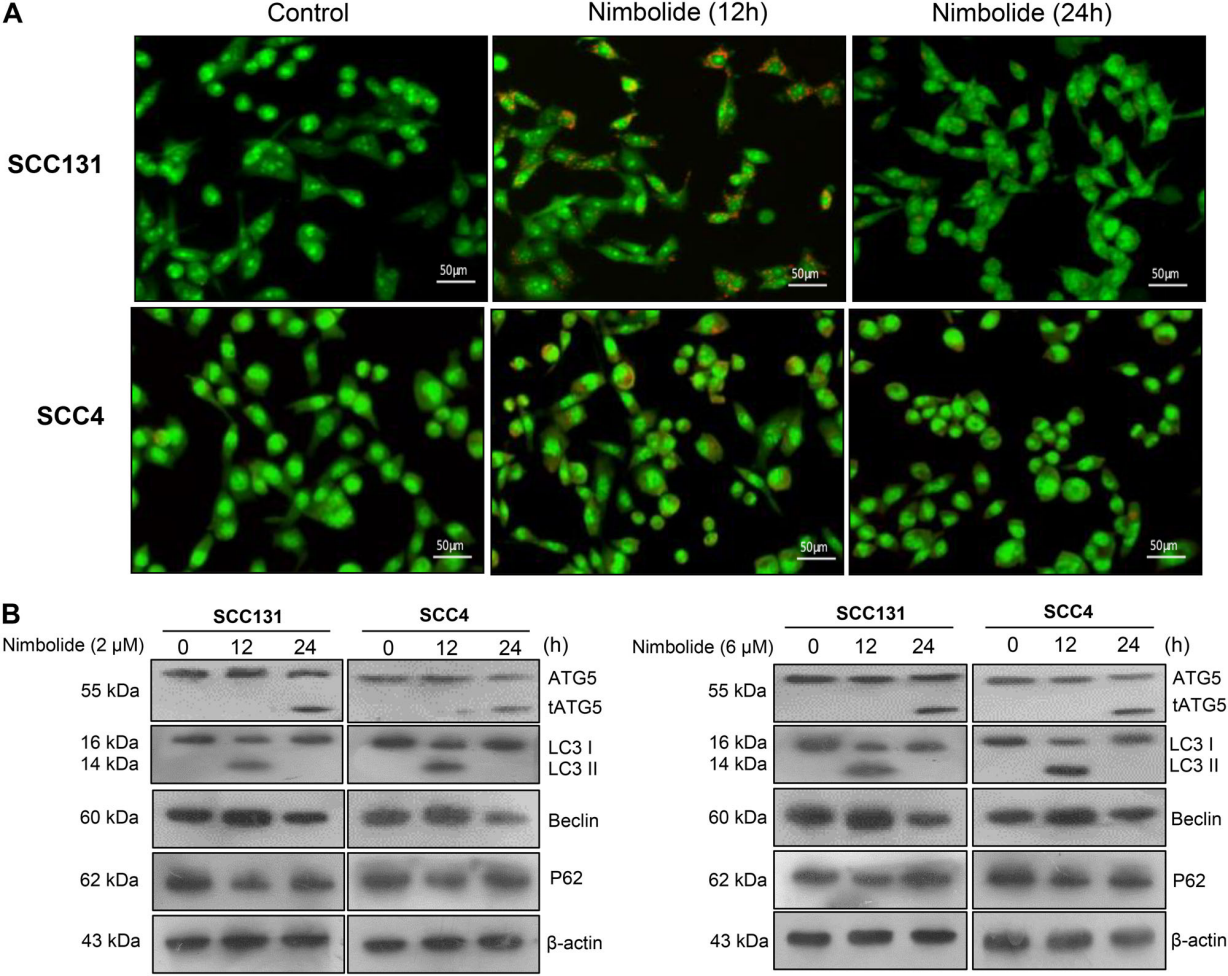
Nimbolide triggers autophagy in oral cancer cells. **a** Cells treated with nimbolide for 12 and 24 h were stained with acridine orange. Representative images of acridine orange-stained acidic vesicular organelles captured by fluorescence microscope (×20) were shown. **b** Western blot showing expressions of LC-3 I/II, ATG5, p62 and Beclin-1 in cells treated with nimbolide for 12 and 24 h. β-actin was used as loading control

Since apoptosis and autophagy are intricately interlinked, we first assessed whether nimbolide induces autophagy in SCC131 and SCC4 oral cancer cells using acridine orange (AO) staining. The protonated form of AO accumulates in autophagolysosomes to form aggregates characterized by orange-red fluorescence. While control cells showed green diffuse pattern of fluorescence, cells treated with nimbolide displayed visible cytoplasmic red punctate fluorescence (Fig. 3a). Immunoblot analysis of LC3, a well-known marker of autophagosome assembly revealed increased conversion of LC3-I to LC3-II in cells treated with nimbolide for 12 h. After 24 h of treatment with nimbolide Beclin-1 expression was decreased with increased expression of p62 associated with presence of truncated ATG5 (24 kDa). Together, these findings suggest that nimbolide induces autophagy (Fig. 3b).

### Nimbolide induces crosstalk between autophagy and apoptosis in SCC131 and SCC4 cells

**Fig. 4:**
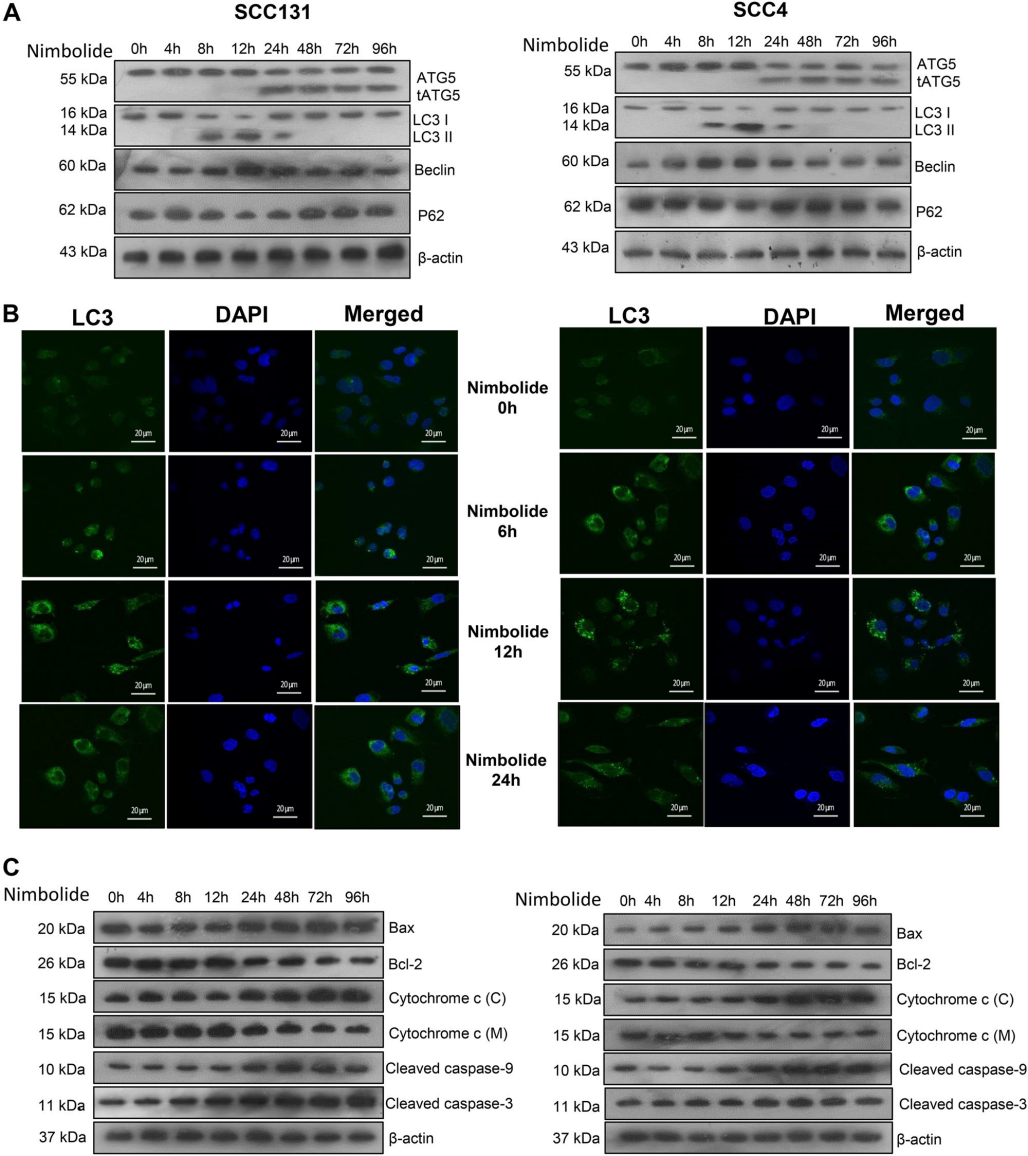
Crosstalk between autophagy and apoptosis upon nimbolide treatment. **a** SCC131 and SCC4 cells were treated with nimbolide from 0 to 96 h. Expression of key molecules involved in autophagy was analysed by western blotting. β-actin was used as loading control. **b** Cells were treated with nimbolide for 6, 12 and 24 h. The LC-3 II puncta formation was viewed and captured by confocal microscope. LC-3 (green) and DAPI (nuclei, blue). Scalebar: 20 μm. **c** Oral cancer cells were treated with nimbolide for 0 to 96 h. Cell lysates were subjected to western blotting with Bax, Bcl-2, cytochrome c, cleaved caspase-9 and -3 antibodies. β-actin was used as loading control

We next sought to investigate the interplay between apoptosis and autophagy following incubation of oral cancer cells with nimbolide. Expression of proteins involved in autophagy and apoptosis were analysed in nimbolide treated cells at various time intervals. We determined the expression of ATG5 and Beclin-1 that function as molecular links between autophagy and apoptosis by immunoblotting. In a time-course experiment, expression of truncated ATG5 first observed at 24 h of nimbolide treatment steadily increased from 24 to 72 h and declined thereafter. Nimbolide treatment induced the conversion of cytosolic LC3-I to autophagosome associated LC3-II from 12 to 24 h (Fig. 4a).

Assessing the LC-3 puncta formation in autophagic vacuoles is a reliable method for monitoring autophagy. The formation of autophagic vacuoles was further confirmed by distribution of LC-3 puncta in nimbolide treated cells using confocal microscopy. Diffuse green fluorescence was observed in control cells whereas significant increase in the characteristic punctate fluorescent patterns was observed in nimbolide treated cells. Autophagosome formation associated with simultaneous increase in the expression of autophagy-related proteins suggests that nimbolide induces autophagy in oral cancer cells (Fig. 4b).

As truncated ATG5 is known to influence apoptosis, we next determined the expression of molecules involved in transducing intrinsic apoptosis at various time intervals. Western blot analysis showed significant increase in the expression of Bax, cytochrome c and cleaved caspase 3 from 24 h till 96 h with simultaneous decrease in the expression of Bcl-2 from 24 to 96 h in nimbolide treated cells. These results imply that nimbolide initially induces autophagy and switches over to apoptosis after 24 h (Fig. 4c).

**Fig. 5:**
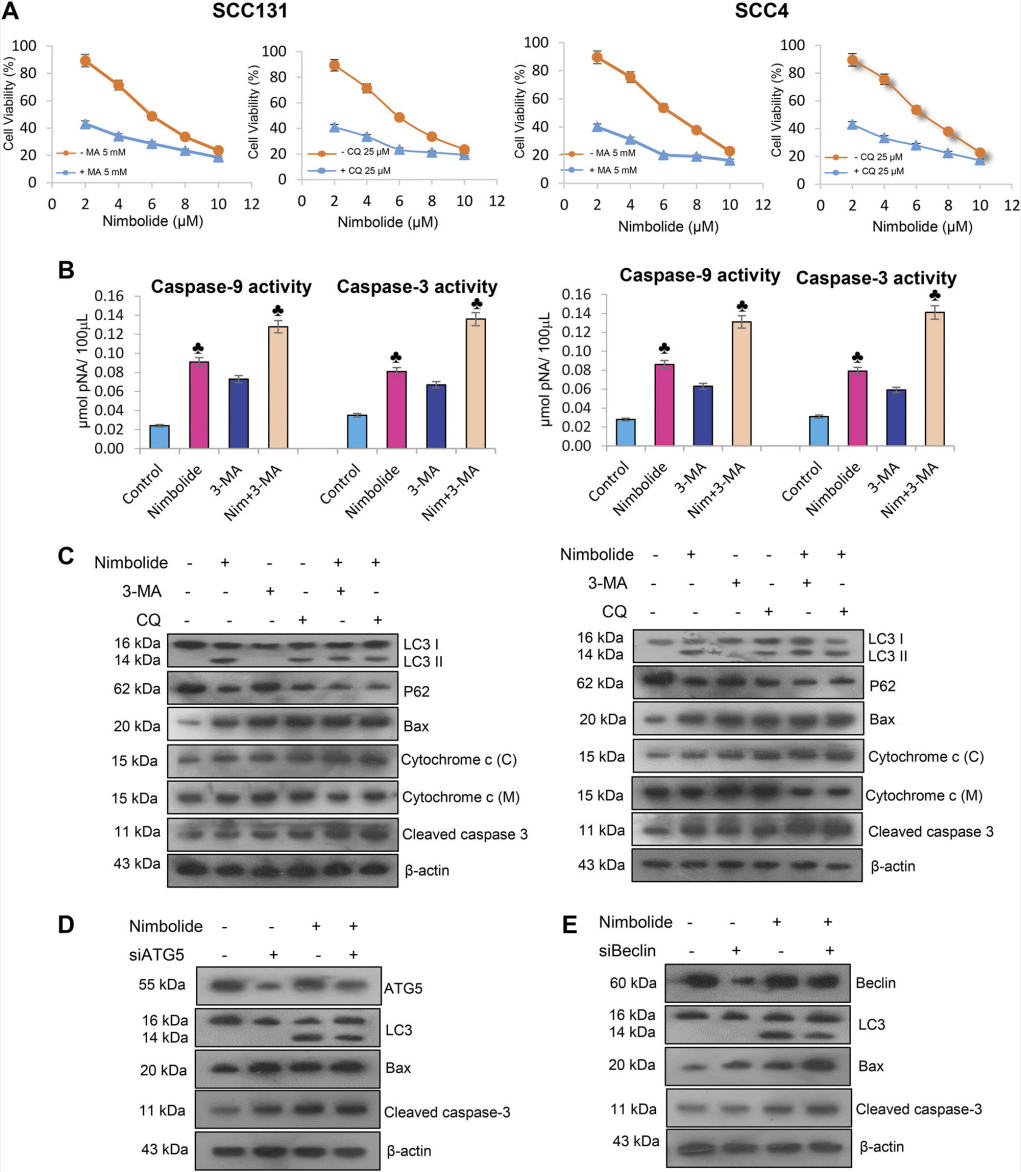
Effect of small molecule inhibitors (SMIs) and gene silencing of autophagy on nimbolide-induced apoptosis and autophagy. **a** Cells were pre-treated with early and late stage autophagy inhibitors, 3-MA or CQ followed by treated with nimbolide for 12 h, and then cell viability was assessed by the MTT assay. **b** Activities of caspase-9 and -3 in cells exposed to 3-MA and CQ in the presence or absence of nimbolide. **c** SCC131 and SCC4 cells were exposed to 3-MA and CQ in the presence or absence of nimbolide. Cell lysates were prepared and subjected to western blot by using antibodies against LC-3, p62, Bax, cytochrome c and cleaved caspase-9 and -3. β-actin was used as loading control. **d** Immunoblot analyses of ATG5, LC-3, Bax and cleaved caspase-3 in control siRNA and ATG5 siRNA transfected SCC4 cells in the absence or presence of nimbolide. **e** Immunoblotting was performed to analyse the expression of ATG5, LC3, Bax, and cleaved caspase-3 in control siRNA and Beclin siRNA transfected SCC4 cells in the absence or presence of nimbolide

To check whether nimbolide induced autophagy is cytoprotective or cytotoxic, untreated and nimbolide treated cells were incubated with early and late stage autophagy inhibitors, 3-methyladenine (3-MA) and chloroquine (CQ) respectively. The growth inhibitory potential of 3-MA and CQ in the presence and absence of nimbolide was tested by MTT assay. Cells treated with 3-MA (5 mM) and CQ (25 µM) for 24 h showed decreased cell viability (Fig. 5a). Moreover, caspase-3/9 activities were increased in the presence of 3-MA and CQ in nimbolide treated cells indicating that autophagy inhibition could facilitate apoptosis and nimbolide induced autophagy is cytoprotective (Fig. 5b). Enhanced expression of Bax, cytosolic cytochrome c and cleaved caspase-3 with decreased expression of LC-3 II, Beclin-1 and ATG5 confirmed the cytoprotective effect of autophagy induced by nimbolide (Fig. 5c).

To further verify the cytoprotective action of autophagy, we silenced Beclin-1 and ATG5 in the presence and absence of nimbolide in SCC4 cells. Cells transfected with Beclin-1 and ATG5 siRNA in the presence of nimbolide showed reduced expression of Beclin-1, ATG5, and LC3-II. Knockdown of Beclin-1 and ATG5 enhanced the expression of Bax and cleaved caspase-3 indicating that autophagy is cytoprotective. These results suggest that autophagy is a cytoprotective mechanism for OSCC cells in the context of nimbolide-induced apoptotic cell death (Fig. 5d, e).

### Nimbolide induces autophagy via inhibition of PI3K pathway in oral cancer cells

**Fig. 6:**
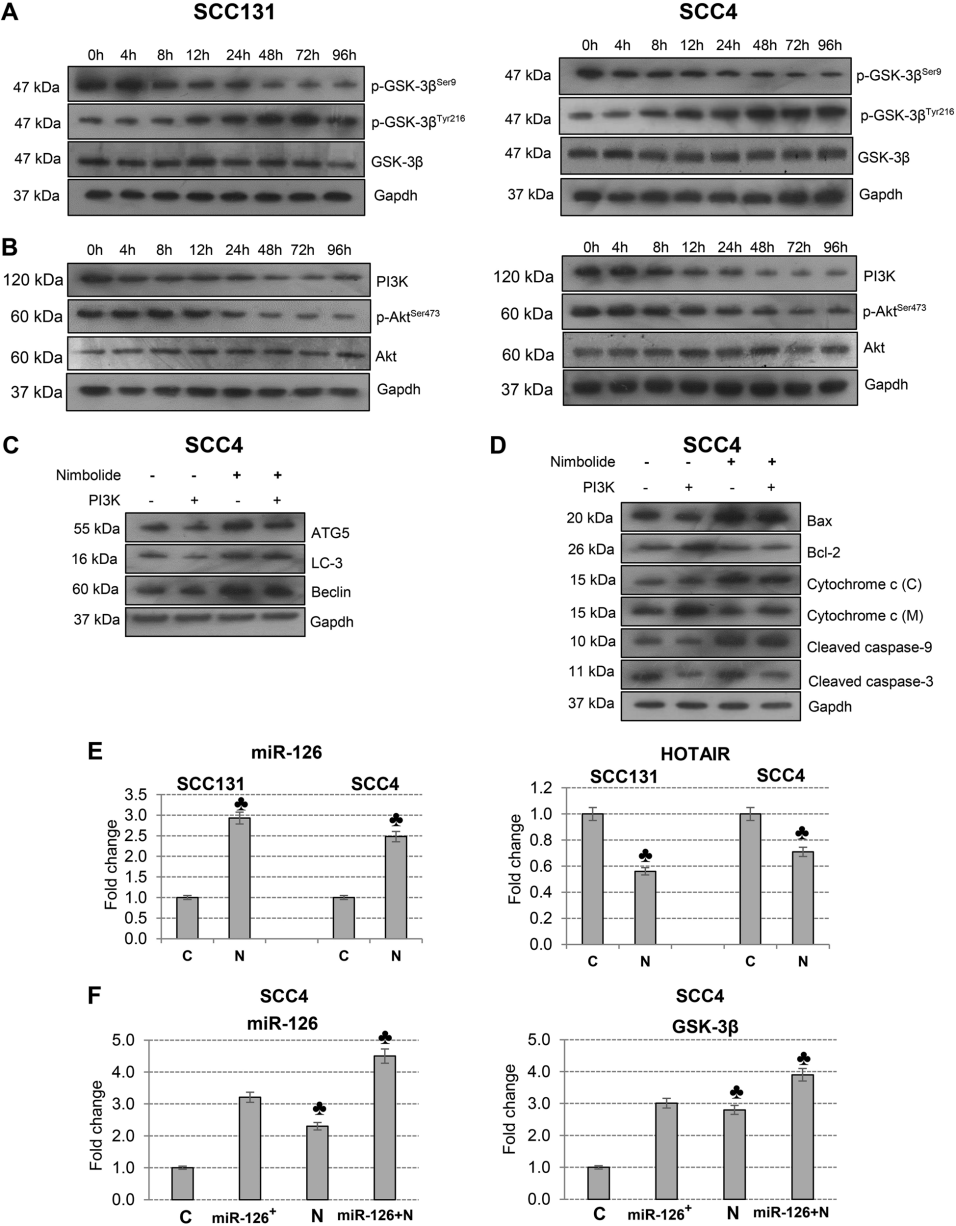
Nimbolide modulates PI3K/Akt/GSK-3β via targeting HOTAIR and miR-126. **a** Western blots showing expression of phosphorylated and unphosphorylated GSK-3β in cells treated with nimbolide for 0 to 96 h. Gapdh was used as loading control. **b** Immunoblot analysis of PI3K, p-Akt^Ser473^, and Akt in cells treated with nimbolide for 0 to 96 h. Gapdh was used as loading control. **c** Immunoblot analysis of molecules involved in autophagy in SCC4 cells transfected with empty plasmid and PI3K plasmid in the absence or presence of nimbolide. Gapdh was used as loading control. **d** Immunoblot analyses of Bax, Bcl-2, cytochrome c, cleaved caspase-9 and -3 in empty plasmid and PI3K plasmid transfected cells in the absence or presence of nimbolide.The data presented are representative of three independent experiments. **e** qRT-PCR analysis of miR-126 and HOTAIR in SCC131 and SCC4 cells. Data are the mean ± SD of three independent experiments. **f** Transcript expression level of miR-126 and GSK-3β in control and miR-126 overexpressed cells in the presence or absence of nimbolide (6 µm) as determined by quantitative RT-PCR. ♣ Significantly different from control (*p* < 0.001)

Since PI3K and GSK-3β are involved in the regulation of autophagy and apoptosis, we investigated the expression of Akt, GSK-3β and their phosphorylation status in a time-dependent manner. Our results revealed that nimbolide treatment significantly decreased the phosphorylation status of Akt from 24 to 96 h with simultaneous enhanced and sustained expression of the p-GSK-3^Thy216^ (active form) from 24 to 96 h in both SCC131 and SCC4 cells (Fig. 6a, b). To explore the mechanism underlying the role of PI3K in nimbolide mediated autophagy and apoptosis, we overexpressed PI3K in SCC4 cells. As shown in Fig. 6c, d, PI3K overexpression induced autophagy by upregulation of ATG5, LC-3 and Beclin with downregulation of the expression of Bax, cytochrome c, cleaved caspase-3 and -9 that was overcome by nimbolide administration. These results suggest that nimbolide mediates apoptosis and autophagy via PI3K and GSK-3β pathways.

### Nimbolide stimulates GSK-3β expression by modulating miR-126 and HOTAIR

In our previous study, we showed that administration of nimbolide to DMBA painted hamsters increased the expression of miR-126, an activator of GSK-3β. Here, we demonstrate that nimbolide treatment significantly increased the expression of miR-126 in oral cancer cells. To strengthen these findings, we overexpressed miR-126 in SCC4 cells. Overexpression of miR-126 upregulated the expression of GSK-3β compared to control cells. Addition of nimbolide to miR-126 overexpressed cells enhanced this effect. Additionally, nimbolide downregulated the expression of Homeobox transcript antisense intergenic RNA (HOTAIR), a lncRNA that inhibits miR-126. Taken together, our results suggest that inhibition of HOTAIR by nimbolide could increase the expression of miR-126 leading to activation of GSK-3β (Fig. 6e, f).

### Nimbolide modulates autophagy and apoptosis via targeting the PI3K/Akt/GSK-3 pathway in the HBP model

We next sought to determine whether nimbolide influences apoptosis/autophagy in the HBP model. Earlier, we demonstrated significant regression of HBP tumors associated with apoptosis induction after 4 weeks of nimbolide treatment^15^. In this study, nimbolide was administered to DMBA painted hamsters at 8 weeks when dysplasia developed and at 12 weeks when SCCs were evident with a view to evaluate the effects on apoptosis/autophagy flux.

**Fig. 7:**
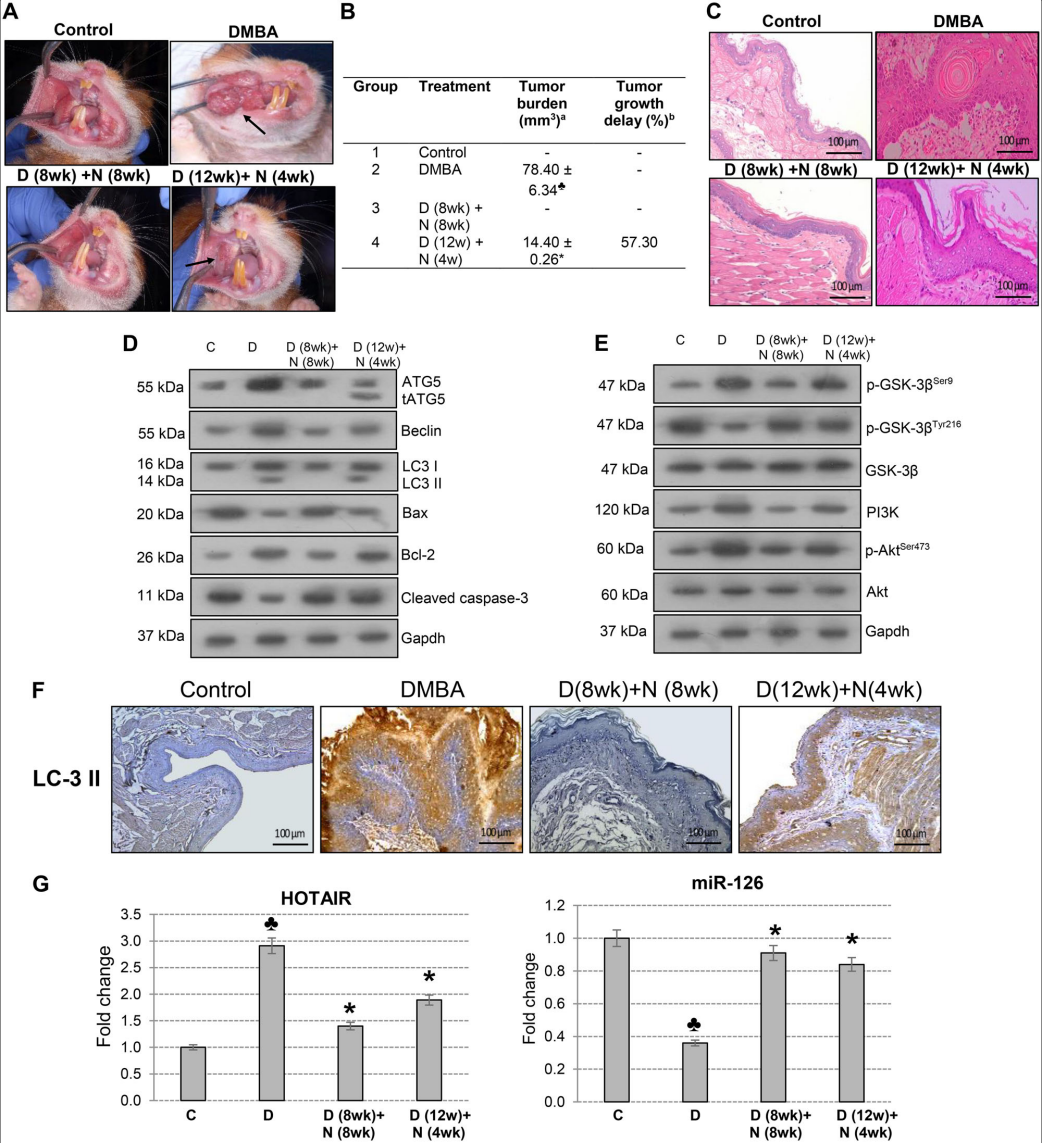
Chemotherapeutic effect of nimbolide in the HBP model (mean ± SD; *n* = 3). **a** Gross appearance of the buccal pouch mucosa. **b** Tumor burden and tumor growth delay in the buccal pouch of hamsters. ^a^Mean tumor burden was calculated by multiplying the mean tumor volume (4/3πr^3^) with the mean number of tumors (*r* = ½ tumor diameter in mm). ^b^Tumor growth delay was calculated by dividing differences in tumor volume (16th week) between DMBA-treated group and chemotherapy group multiplied by 100. **c** H&E stained regions of buccal pouch mucosa of control and experimental animals. (20×). **d** Expression of proteins involved in autophagy and apoptosis were analysed by western blotting. Gapdh was used as loading control. **e** Immunoblotting was performed to analyse the expression of PI3K, Akt, and GSK-3β. Gapdh was used as loading control. **f** Immunohistochemical analysis of LC-3 in hamsters treated with nimbolide for 8 and 4 weeks. **g** Quantitative RT-PCR analysis of HOTAIR and miR-126. The fold change in transcript expression for each gene was determined using the 2^−ΔΔCt^ method. Data are the mean ± SD of three separate experiments. Statistical significance was determined by the Mann–Whitney test (*p* < 0.05). ♣ Significantly different from control (*p* < 0.05). * Significantly different from DMBA-treated group (*p* < 0.05). **c**, Control; **d**, Hamsters painted with DMBA for 12 weeks; D (8 wk) + N (8 wk), Hamsters painted with DMBA for 8 weeks followed by treatment with nimbolide from the 8th to the 16th week; **d** (12 wk) + N (4 wk), Hamsters painted with DMBA for 12 weeks followed by nimbolide administration from 12th to 16th week

Although nimbolide intervention at both time points exerted chemotherapeutic effects, early intervention at 8 weeks was more significant. As illustrated in Fig. 7, administration of nimbolide after 8 weeks of DMBA painting arrested tumor growth associated with induction of apoptosis and inhibition of autophagy as evidenced by a significant increase in Bax and cleaved caspase-9 and -3 with downregulation of Bcl-2, Beclin, ATG5, and LC-3. On the other hand, when nimbolide was administered after 12 weeks of DMBA painting, there was significant tumor growth delay (57.30%) and reduced tumor burden compared to hamsters painted with DMBA alone. Interestingly, presence of tATG5 was observed in addition to upregulation of proapoptotic molecules indicating a switchover to apoptosis at this time point. Furthermore, nimbolide administration at both time points abrogated PI3K/Akt/GSK-3β signalling with more significant effects when the intervention was early. Together, these data provide compelling evidence that nimbolide sensitizes tumor cells to apoptosis by inhibiting cytoprotective autophagy.

### Expression of molecules involved in autophagy and apoptosis during progression of oral carcinomas

**Fig. 8:**
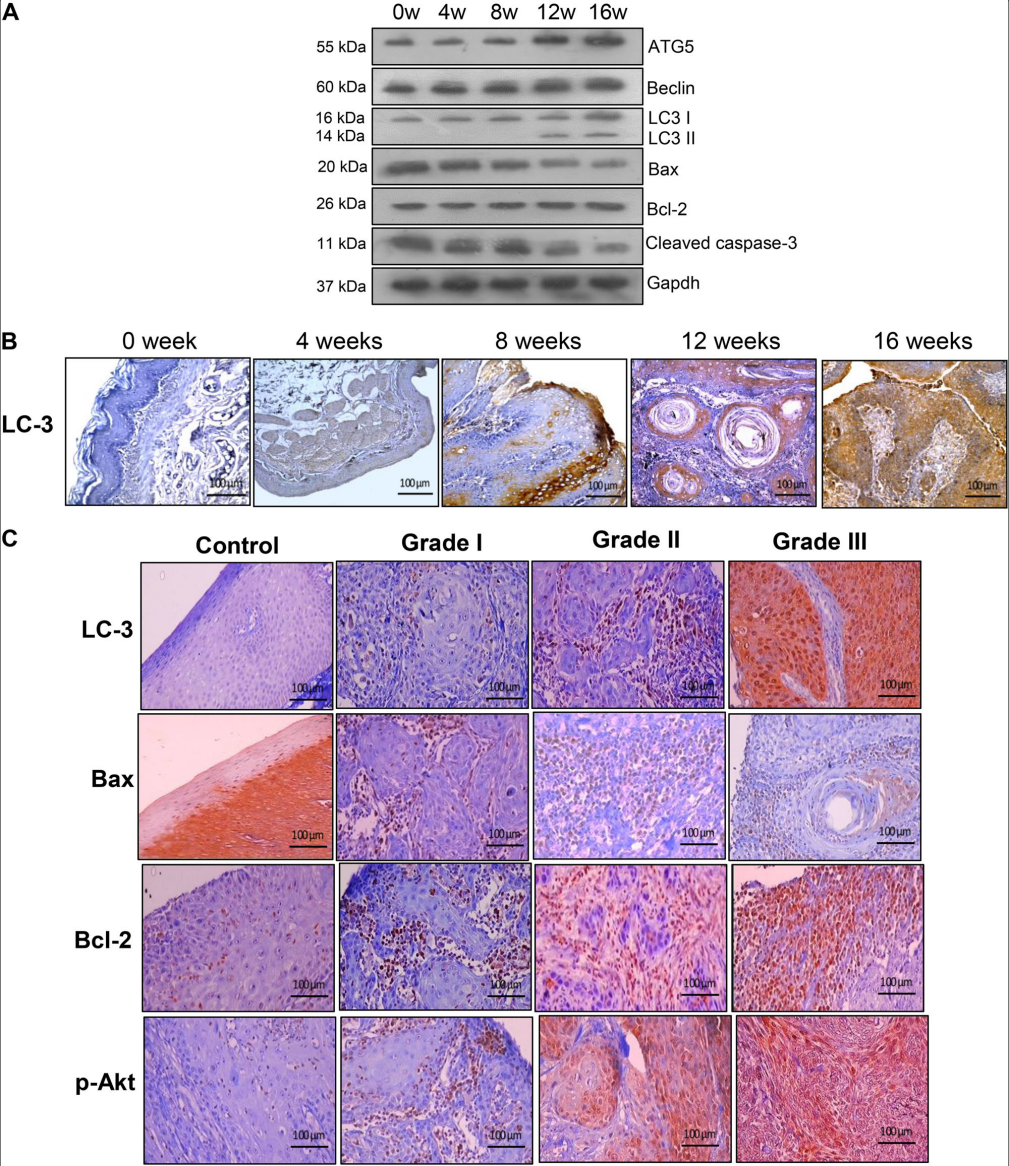
The expression of key molecules involved in autophagy and apoptosis during sequential progression of oral carcinogenesis (mean ± SD; *n* = 3). **a** Immunoblotting was performed to analyse the expression of ATG5, Beclin, LC-3, Bax, Bcl-2, and Cleaved caspase-3 during sequential progression of HBP carcinomas. β-actin was used as loading control. **b** Representative photomicrographs of immunohistochemical staining of LC-3 during sequential progression of HBP carcinomas (×40). **c** Representative photomicrographs of immunohistochemical staining of LC-3, Bax, Bcl-2, and p-Akt^Ser473^ in control and different histological grades of human oral cancer tissues (×40). 0 w, 0 week; 4 w, 4 weeks; 8 w, 8 weeks; 12 w, 12 weeks; 16 w, 16 weeks

Finally, we evaluated the expression of key molecules involved in apoptosis and autophagy during different stages of HBP carcinogenesis and different histological grades of human OSCC. We found a sustained increase in Bcl-2, Beclin-1, ATG5,LC-3 and p-Akt^Ser473^ associated with decreased expression of Bax and cleaved caspase-3 during progression of normal epithelium through hyperplasia, dysplasia and well-differentiated squamous cell carcinoma of the hamster buccal pouch. Similarly, the expression of Bcl-2, LC-3 and p-Akt^Ser473^ was significantly overexpressed with downregulation of Bax in grade II and grade III human OSCC, compared to grade I and grade II. These results confirm the similarity in the expression pattern of apoptosis/autophagy proteins in hamsters and human OSCCs (Fig. 8).

## Discussion

There is substantial evidence to indicate switching of cell fate from autophagy to apoptosis depending on the severity of stress^33–36^. Concurrent regulation of autophagy and apoptosis is therefore regarded as a promising strategy for the development of anticancer agents and may improve therapeutic outcome in cancer. Several chemotherapeutic agents and phytochemicals have been identified in preclinical and clinical studies that abrogate autophagy and induce apoptosis of tumor cells^36–39^. We report for the first time that nimbolide initially induces cytoprotective autophagy with subsequent switchover to apoptosis in oral cancer cells as well as in the HBP model. We further show that this flux is mediated through regulation of proteins and signalling pathways that control these two cellular processes. Additionally, we have documented changes in the expression of key molecules involved in the two forms of PCD during the sequential progression of hamster and human OSCCs that may have implications for therapeutic intervention.

The proapoptotic effects of nimbolide have been extensively documented by us as well as others^11–13,15,19–23^. In the present study, nimbolide induced stereotypical changes in SCC131 and SCC4 oral cancer cells characteristic of both apoptosis and autophagy. Nimbolide transduced apoptosis by the mitochondrial pathway as evidenced by increased Bax/Bcl-2 ratio, efflux of cytochrome c into the cytosol and caspase activation. Nimbolide also induced classic hallmarks of autophagy including accumulation of acidic vesicles, increased expression of Beclin-1 and ATG5, conversion of LC3-I to LC3-II as well as degradation of p62, an adaptor protein that recognizes and binds to proteins targeted for degradation.

Results from our time-course experiments revealed enhanced expression of Beclin-I and LC-3 II with reduced expression of p62 from 4 to 12 h and presence of truncated ATG5 after 12 h of nimbolide treatment. On the other hand, the expression of apoptosis markers was increased from 24 h to 96 h clearly indicating that nimbolide induces autophagy as an early event and then switches over to apoptosis. Inhibition of autophagy by small molecule inhibitors (SMIs) as well as siRNA silencing of ATG5 and Beclin-1 enhanced apoptosis confirming that nimbolide induced autophagy-dependent apoptosis in oral cancer cells. Recently, Liu et al.^36^ reported that inhibition of autophagy by ATG5 knockdown enhanced apoptosis of colon cancer cells upon ginkgolic acid treatment. Likewise, the autophagy inhibitor CQ was found to increase the proapoptotic ability of Licarin A in non-small cell lung cancer cells^39^. Methanolic extract of neem seed containing nimbolide was reported to induce apoptosis of human osteosarcoma cells via inhibition of cytoprotective autophagy^40^. These findings support the tenet that following treatment with antiproliferative agents, cells temporarily undergo autophagy as a prosurvival mechanism that is subsequently subverted to favor apoptosis. We believe that mutual interaction between proteins that regulate apoptosis and autophagy mediate the switchover. While ATG5 and Beclin-1, indispensable for autophagy also stimulate apoptosis, BCL-2, an autophagy inhibitor is a dual regulator of both the pathways. Mounting evidence indicates that BCL2-Beclin-1 interactions are largely responsible for the autophagy-apoptosis flux^41–43^. Caspases are known to function as molecular switches between the two forms of PCD by cleaving ATG proteins. Conversely, the truncated proteins activate caspase-mediated apoptosis^44,45^.

Several lines of evidence have revealed an overlap in the signalling pathways governing apoptosis and autophagy that orchestrate distinct modes of PCD often with opposing outcomes. In particular, the PI3K/Akt signalling axis plays a pivotal role in mediating the crosstalk between apoptosis and autophagy^46–54^. Once activated, Akt inactivates several proapoptotic proteins as well as GSK-3β, a central hub in oncogenic signalling^49,50^. Marchand et al.^51^ reported that inhibition of GSK-3β induces prosurvival autophagy/lysosomal network in human pancreatic cancer cells. Natural products that target the PI3K/Akt/GSK-3β pathway promote autophagy to sensitize cancer cells to apoptosis^52^. Pterostilbene, a naturally occurring phytoalexin induced autophagy and apoptosis of human oral cancer cells by inhibiting p-Akt^53^. Previously, we demonstrated that nimbolide induces apoptosis of HBP carcinomas by abrogating the PI3K/Akt pathway with consequent activation of GSK-3β^15^. Here, we show that nimbolide negatively regulates activation of PI3K/Akt in oral cancer cells by inhibiting phosphorylation of Akt at Ser473 with consequent increase in p-GSK3β^Tyr216^, the active form of GSK3β that inhibits autophagy. Nimbolide-mediated ROS generation was shown to inhibit proliferation and metastasis of pancreatic cells via blockade of PI3K/AKT/mTOR/ERK signalling and activation of mitochondrial apoptosis^21^. Interestingly, azadirachtin, another neem limonoid, was demonstrated to inhibit proliferation of *Spodoptera litura* insect cells by first triggering autophagy through dysregulation of the PI3K/Akt/mTOR signalling axis and then stimulating apoptosis via truncation of ATG5^54^. Together, these results reveal that neem limonoids exert their anticancer effects via modulation of PI3K/Akt signalling.

Of late, ncRNAs have gained increasing attention as novel players that modulate oncogenic signalling pathways in oral cancer^55^. HOTAIR, a long non-coding RNA overexpressed in most human cancers including OSCCs activates PI3K/Akt signalling by inactivating the tumor suppressor PTEN, an inhibitor of PI3K^56^. On the other, miR-126 that negatively regulates the PI3K/Akt pathway by activating GSK3β is downregulated in OSCC cell lines and tissues^15^. Downregulation of HOTAIR, a competing endogenous RNA (ceRNA) that sponges miR-126 may be a major contributor to inactivation of PI3K/Akt/GSK3 signalling by nimbolide. An interplay between HOTAIR and miR-126 observed in osteosarcoma cells by Li et al.^57^ substantiate these findings. Recently, nimbolide was shown to promote H3K27 acetylation by inhibiting HDAC2, eventually inducing autophagy-driven apoptosis of breast cancer cells^27^. In an earlier study, we reported that administration of nimbolide to hamsters painted with DMBA significantly inhibited HDAC1 that plays a critical role in cell proliferation and apoptosis evasion^17^. Together, these studies unveil the modulatory effects of nimbolide on the epigenome.

The chemopreventive efficacy of nimbolide is well established in the HBP model^16,17^. Although we have reported the chemotherapeutic effects of nimbolide in an earlier study^15^, here we demonstrate that the therapeutic efficacy is dependent on the duration of exposure as well the stage in the natural history of tumor progression. Quite understandably, nimbolide was more efficacious when administered after 8 weeks of DMBA painting when dysplastic lesions appear and for a longer duration of 8 weeks. We also provide evidence to show that nimbolide exerts modulatory effects on the expression of molecules involved in the regulation of apoptosis and autophagy potentiating the findings from the cell-based assays. Analysis of BCL2, Bax, and LC-3, key markers of apoptosis and autophagy as well as p-Akt^Ser473^ during the sequential progression of hamster and human OSCC revealed a gradual evolution to a pro-autophagic and antiapoptotic phenotype that could confer a survival advantage to tumors. Previously, we reported a correlation between BCL2 expression and OSCC progression^58^. High expression of LC-3, one of the most reliable markers of autophagy was closely associated with TNM staging and lymph node metastasis^59^. Elevated LC3 expression, an indicator of poor prognosis in patients with OSCC, correlated with poor survival^60^. Similarly, a significant association between p-Akt ^Ser473^ overexpression and adverse prognosis of OSCC reported in literature is consistent with the sustained increase in p-Akt^Ser473^ expression during progression of human and hamster OSCC^61,62^.

In summary, the results of the present study provide insights into the molecular mechanisms by which nimbolide augments apoptosis by overcoming the shielding effects of cytoprotective autophagy through modulation of the PI3K/Akt signalling cascade by altering the phosphorylation status of Akt and GSK-3β as well as the ncRNAs miR-26 and HOTAIR. Given the prevalence and poor prognosis of OSCC and the adverse effects of current treatments, development of phytochemicals such as nimbolide that target the complex interaction between proteins and ncRNAs that regulate the autophagy/apoptosis flux is of paramount importance. This study has also reiterated the validity of using the hamster model as a paradigm for oral oncogenesis and chemointervention.

## Materials and Methods

### Reagents and antibodies

Acrylamide, AO, bovine serum albumin (BSA), bromophenol blue, CQ, 4,6-diamidino-2-phenylindol (DAPI), DMBA, ethidium bromide, JC-1 iodide, 3-methyladenine (3-MA), 2-mercaptoethanol, 3-(4,5-dimethylthiazol-2-yl)-2,5-diphenyl tetrazolium bromide (MTT), sodium dodecyl sulphate (SDS), N,N,N’,N’-tetramethylene diamine (TEMED) and Trizol were acquired from Sigma Chemical Company, St. Louis, MO, USA. Power SYBR^®^ Green PCR master mix was obtained from Applied Biosystems, California, USA. Antibodies for Akt, β-actin, β-catenin, cleaved caspase-3, cleaved caspase-9, cytochrome c, GSK-3β, p-GSK-3β^Ser9^, p-GSK-3β^Tyr216^, PI3K, and Gapdh were purchased from Santa Cruz Biotechnology, USA. Antibodies for ATG5, Bax, Bcl-2, Beclin-1, Histone H2B, LC-3, p-Akt^Ser473^, p-β-catenin^Ser33,Ser37,Thr41^, and p-β-catenin^Ser552^ as well as ELISA kits were from Cell Signaling Technology, USA. Alexafluor-488 conjugated anti-rabbit antibody was obtained from Molecular Probes, Inc. (Eugene, OR, USA). Annexin V-FITC, propidium iodide (PI) kit and p62 antibody were purchased from BD Biosciences (San Diego, CA). Nimbolide was obtained from M/s Asthagiri Herbal Research Foundation, Chennai, India. FuGENE transfection reagent was procured from Promega. Oligonucleotide primers were purchased from Sigma Genosys, San Ramon, USA. All other reagents used were of analytical grade.

### Cell culture

SCC131 cells were cultured in DMEM basal medium supplemented with 10% fetal bovine serum (FBS; Gibco) and antibiotics. SCC4 cells were grown in DMEM/F12 supplemented with 10% FBS, 2 mM glutamine and 0.4 μg/ml hydrocortisone. Cells were maintained as monolayer cultures in a humidified atmosphere of 5% CO_2_ at 37 °C. Exponentially growing cells were used for all the experiments.

### Animals and diet

The experiments were carried out using 8–10 weeks old male Syrian hamsters weighing between 100 and 110 g, procured from the National Centre for Laboratory Animal Sciences (NCLAS), Hyderabad, India. The animals were accommodated six to a polypropylene cage and provided with standard pellet diet (Sai Enterprisei, Chennai, India) and water ad libitum. The animals were maintained in a controlled environment under standard conditions of temperature and humidity with an alternating 12 h light/dark cycle in accordance with the guidelines of the Indian Council of Medical Research. All experimental procedures were approved by the Institutional Animal Ethics Committee, Annamalai University and conducted according to the guidelines by the Committee for the Purpose of Control and Supervision on Experiments on Animals (CPCSEA).

### Experimental design

#### Experiment 1

The animals were randomized into experimental and control groups and divided into 5 groups of 6 animals each. Animals in group 1 received basal diet alone and served as control. The right buccal pouches of hamsters in the experimental groups 2–5 were painted with a 0.5% solution of DMBA in liquid paraffin, three times per week for 4, 8, 12 and 16 weeks, respectively^63,64^. At the end of 0, 4, 8, 12 and 16 weeks of DMBA application, animals in the respective groups were sacrificed by cervical dislocation after an overnight fast. Before an animal was killed, the right pouch was grossly inspected to evaluate premalignant lesions and tumor development. A portion of the buccal pouch tissue was immediately frozen in liquid nitrogen for subsequent RNA extraction, while another portion was processed using lysis buffer for western blot analysis.

#### Experiment 2

The animals were divided into 4 groups of 6 animals each. Hamsters in group 1 served as untreated control. The right buccal pouches of hamsters in group 2 were painted with 0.5% DMBA for 12 weeks followed by a basal diet up to the 16th week. To test the chemotherapeutic effect of nimbolide, we treated hamsters with nimbolide at two different time points. Animals in group 3 were painted with DMBA for 8 weeks followed by treatment with nimbolide (100 µg/kg bw) from the 8th week when dysplasia was observed until the 16th week. In group 4, hamsters were painted with DMBA as in group 2, followed by intragastric administration of nimbolide (100 µg/kg bw) from the 12th week when SCC was evident and continued until the end of the experimental period^15^.

### Patients and tissue samples

A total of one hundred and twenty (*n* = 120) human oral cancer and control samples were used for the study. This includes tissue microarray (TMA, OR802, and OR601a) from US Biomax (*n* = 81) and freshly collected human oral tumor and control samples (*n* = 39) from Rajah Muthiah Dental College and Hospital, Annamalai University. These samples were collected after obtaining informed consent from the patients, and the use of human samples was approved by the Human Institutional Ethics Committee (IHEC). The collected samples were stored in buffered formalin. Staging of the oral cancer samples was conducted according to American Joint Committee on Cancer (AJCC)/International Union against Cancer (UICC).

### Cell cytotoxicity assay

Cytotoxicity was assessed by the MTT assay based on the reduction of MTT by mitochondrial dehydrogenases of viable cells to a purple formazon product^65^. Cells were seeded in a 96-well plate (Tarsons, India) at a density of 1 × 10^5^ cells per well. After overnight growth, cells were treated with various concentrations of nimbolide (0–10 μM) and incubated for 24 h. In addition, SCC131 and SCC4 cells individually pre-treated with 3-MA (10 mM) and CQ (25 µM) followed by treatment with or without nimbolide for 24 h. 10 μL of MTT was added to each well, and the plates were incubated for 3 h at 37 °C. Then 100 μl of acidified isopropanol was added to dissolve MTT-formazon product and the absorbance was measured at 595 nm in a microtiter plate reader.

### Assessment of nuclear morphology

Characteristic apoptotic morphological changes were evaluated by fluorescence microscopy using DAPI staining. SCC131 and SCC4 cells were seeded at a density of 2 × 10^5^ into a 12-well plate. After 24 h of nimbolide treatment, cells were fixed in ice-cold methanol (−20 °C) for 15 min and stained with DNA-specific fluorochrome DAPI (1 μg/ml) for 30 min in the dark. The cells were immediately washed with PBS, viewed and photographed using Nikon inverted fluorescent microscope (TE-Eclipse 300).

### Analysis of mitochondrial transmembrane potential

The changes in the mitochondrial transmembrane potential (ΔψM) were determined using JC-1, a fluorescent carbocyanine dye, which accumulates in the mitochondrial membrane as a monomer or dimer depending on the mitochondrial membrane potential^66^. Briefly, cells were plated at a seeding density of 2 × 10^5^ cells/well in a 12-well plate. After 24 h of treatment with nimbolide, cells were incubated with 5 µM of JC-1 for 30 min at room temperature in the dark. The presence of JC-1 monomers or dimers were examined under a fluorescence microscope using filter pairs of 530 nm/590 nm (dimers) and 485 nm/ 538 nm (monomers).

### Detection of autophagic vesicles by AO staining

Briefly, cells were plated at a seeding density of 2 × 10^5^ cells/well in a 12-well plate. After treatment with nimbolide for 12 and 24 h, cells were incubated with AO for 30 min. Analysis was performed by fluorescence microscopy using 490-nm band-pass blue excitation filters and a 515-nm long-pass barrier filter. Depending on their acidity, autophagic lysosomes appeared as orange/red fluorescent cytoplasmic vesicles, while cytoplasm and nucleolus were green.

### Cell cycle analysis

Cell cycle distribution and measurement of the percentage of apoptotic cells were performed by flow cytometry (FACS Aria III, BD Biosciences). SCC131 and SCC4 cells were plated in six-well plates at a density of 5 × 10^6^ cells/well. After 24 h of treatment, cells were harvested by trypsinization and washed twice with PBS. Cells were then gently fixed with 70% ice-cold ethanol at −20 °C for 1 h and resuspended in PBS containing 0.5 μg/ml RNase, and incubated at 37 °C for 30 min. Following this, cells were stained with 50 μg/ml propidium iodide for 10 min and the DNA content was analysed on a flow cytometer.

### Annexin V assay

Induction of apoptosis in oral cancer cells upon nimbolide treatment was tested through flow cytometry analysis. SCC131 and SCC4 cells were seeded in a 6-well plate with 5 × 10^4^ cells in each well. After 24 h of treatment with nimbolide (2 and 6 µM), cells were harvested by trypsinization and washed twice with ice-cold PBS. Cells were then stained with annexin-FITC/PI using annexin staining kit as per the manufacturer’s instructions. At the end of the assay, cells were subjected to flow cytometry to determine percent of apoptotic cells. A minimum of 20,000 cells were collected for each measurement.

### Transfection

The siRNA-ATG5 and siRNA-Beclin were purchased from Sigma Chemical Company, St. Louis, MO, USA. PI3K (#16643) plasmid was obtained from Addgene, USA. The integrity of the construct was confirmed by DNA sequencing. Transfection was performed using FuGENE transfection reagent according to the manufacturer’s instructions. After 8 h, the media was renewed followed by treatment with nimbolide for 24 h. At the end of 48 h, RNA and proteins were isolated from the cells for further analysis.

### Confocal microscopy

Cells grown on coverslips were treated with nimbolide at various time points and then fixed with 4% ice-cold paraformaldehyde for 20 min and permeabilized with an acetone methanol mixture (1:3) for 10 min at room temperature. After blocking with 3% BSA for 2 h, the cells were incubated with LC-3 antibody at 4 °C overnight. Next day, the cells were washed three times with Tris-buffered saline (TBS) buffer (50 mM Tris-HCl, pH 7.5, 150 mM NaCl) and incubated with Alexa Fluor 488-conjugated secondary antibody for 1 h at room temperature. The nuclei were stained with DAPI in anti-fade medium (Life Technologies, Grand Island, New York, USA). Fluorescence images were captured using a fluorescence microscope (Model IX81, Olympus, Singapore).

### Assay of caspase-3 and caspase-9 activities

The activities of caspases were assayed using caspase-3 (Sigma, St. Louis MO, USA) and caspase-9 (Calbiochem, USA) colorimetric assay kits according to the manufacturer’s instructions. The assays are based on the hydrolysis of the peptide substrate acetyl-Asp-Glu-Val-Asp-nitroanilide (Ac-DEVD-pNA) by caspase-3, and Leu-Glu-His-Asp-nitroanilide (LEHD-pNA) by caspase-9 and subsequent release of the chromophore p-nitroaniline (pNA). The concentration of pNA released from the substrate was calculated from the absorbance values at 405 nm.

### Quantitative real-time RT-PCR (qRT-PCR) for mRNA, microRNA and lncRNA expression analysis

SCC131 and SCC4 cells grown in 60 mm petri dishes were treated with nimbolide for 24 h. Following treatment, cells were washed with ice-cold PBS and RNA was isolated and cDNA constructed as described previously^15,67^. The cDNA was stored at –80 °C until further use.

MicroRNA was isolated from tissues using miRNeasy minikit method (Qiagen) according to the manufacturer’s protocol and quantified using a Biophotometer (Eppendorf). cDNA was synthesized using NCode^TM^ VILO^TM^ miRNA cDNA Synthesis Kit (Invitrogen).

Quantitative RT-PCR was performed in a StepOne Plus thermocycler (Applied Biosystems) using Power SYBR Green. To the 1 × PCR master mix, 2.5 μl of the cDNA was added in a total volume of 20 μl. The PCR conditions were as follows: 95 °C for 5 min, 40 cycles of 30 s at 95 °C, 30 s at 52–60 °C (based on the target) and 60 s at 72 °C. Relative quantitative fold change was calculated using the comparative Ct method.

### Western blot analysis

Cells/tissues were washed three times with PBS and lysed in a RIPA lysis buffer. The cell lysates were centrifuged at 14,000 rpm for 15 min. Nuclear, cytoplasmic and mitochondrial fractions were prepared as described previously^15^. Total protein content of the whole cell lysate, cytosolic, nuclear and mitochondrial extracts were determined by the method of Bradford^68^. The protein samples were electrophoresed on SDS-PAGE, the resolved proteins transferred to PVDF membraneand probed with corresponding primary and secondary antibodies as described previously^15^. Afterextensive washes with high and low salt buffers, the immunoreactive proteins were visualized using enhanced chemiluminescence (ECL) detection reagents (Sigma). Densitometry was performed on IISP flatbed scanner and quantitated with Total Lab 1.11 software.

### Immunohistochemistry (IHC)

The paraffin-embedded tissue sections were deparaffinized using xylene and dehydrated with graded alcohol and followed by PBS wash. The slides were incubated in citrate buffer (pH 6.0) for antigen retrieval using wet autoclaving method. The sections were allowed to cool to room temperature. The sections were treated for 25 min with 3% H_2_O_2_ in 1X TBST to inhibit endogenous peroxidase activity. After blocking in 1% BSA-PBS for 45 min, the sections were incubated overnight in primary antibody prepared at a dilution appropriate for each of the proteins in blocking buffer at 4 °C overnight. The slides were washed with TBS and then incubated with biotin-labeled secondary antibody followed by streptavidin-biotin-peroxidase (Dako, Carpinteria, CA, USA) for 30 min each at room temperature. The immunoprecipitate was visualized by treating with 3,3 -diaminobenzidine and counterstaining with hematoxylin. The tissues were then photographed using an Inverted Fluorescence Microscope (Leica Microsystem Vertrieb GmbH, Wetzler, Germany) attached with digital camera DFC295.

### Statistical analysis

Cytotoxicity data are presented as mean percentages of control ± S.D and linear regression analysis was used to calculate IC_50_ values. Densitometric analysis data for qPCR and western blot were analysed using Mann–Whitney test (StatsDirect, United Kingdom). A probability value of less than 0.05 was considered significant.
